# Sediment Contaminants and Infauna Associated with Recreational Boating Structures in a Multi-Use Marine Park

**DOI:** 10.1371/journal.pone.0130537

**Published:** 2015-06-18

**Authors:** Vivian X. Y. Sim, Katherine A. Dafforn, Stuart L. Simpson, Brendan P. Kelaher, Emma L. Johnston

**Affiliations:** 1 Evolution and Ecology Research Centre, School of Biological, Earth and Environmental Sciences, University of New South Wales, Sydney, Australia; 2 Sydney Institute of Marine Sciences, Mosman, Australia; 3 Centre for Environmental Contaminants Research, CSIRO Land and Water, Locked Bag 2007, Sydney, Australia; 4 National Marine Science Centre and Centre for Coastal Biogeochemistry Research, School of Environment, Science and Engineering, Southern Cross University, Coffs Harbour, Australia; Institut Maurice Lamontagne, CANADA

## Abstract

Multi-use marine parks achieve conservation through spatial management of activities. Zoning of marine parks in New South Wales, Australia, includes high conservation areas and special purpose zones (SPZ) where maritime activities are concentrated. Although such measures geographically constrain anthropogenic impacts, we have limited understanding of potential ecological effects. We assessed sediment communities and contaminants adjacent to boating infrastructure (boat ramps, jetties and a marina) in a SPZ from the Clyde Estuary in Batemans Marine Park. Metal concentrations and fines content were elevated at boating structures compared to reference sites. Species richness was higher at sites with boating structures, where capitellid polychaetes and nematodes dominated the communities. Changes associated with boating structures were localised and did not extend beyond breakwalls or to reference sites outside the SPZ. The study highlights the benefits of appropriate zoning in a multi-use marine park and the potential to minimise stress on pristine areas through the application of spatial management.

## Introduction

Many estuaries are extensively modified by anthropogenic activities and as a result the resident aquatic communities can be exposed to a range of stressors [1,2]. Urban, industrial and agricultural developments occurring in or adjacent to estuaries alter physical conditions and are often a source of contaminants [3,4]. These changes in physico-chemical conditions have the potential to alter the structure of aquatic communities [5–8]. With approximately 60% of the global human population residing within 100 km of the coast [9], and populations in the coastal zone predicted to continue increasing, the associated development will intensify stress on these already modified systems [1]. This is of particular concern since estuarine ecosystems provide crucial habitat for a diverse range of flora and fauna [10].

Multi-use marine parks that include estuarine areas have been established in Australia, and globally, as a conservation tool to minimise impacts from anthropogenic activities [11]. The zoning of multi-use marine parks provides an opportunity to balance conservation goals with socio-economic interests [12] with spatial management that geographically limits damaging coastal development. By concentrating potentially harmful human activities to geographically explicit areas, it may be possible to maintain commercial and recreational activities in a multi-use marine park while conserving more pristine areas. Over time, this strategy may result in improved estuarine ecosystem function by effective protection of critical habitats (e.g. seagrass). Such positive conservation outcomes will depend of the extent to which impacts from development can be contained, which can be difficult to assess in dynamic aquatic environments [13].

Boating infrastructure, such as jetties, boat ramps, marinas and slipways, represent a tangible threat to estuarine environments that could be concentrated into special purpose zones in a multi-use marine park to constrain the spatial extent of their impacts. Boating infrastructure adds dense networks of artificial structures that significantly modify the surrounding environment [14–17]. The hard structures introduce shading and alter the natural flow regime [15,18], which can affect the physico-chemical properties of the surrounding habitat and the water column transport of propagules and sediments [19,20]. Boating infrastructure and associated vessels can also introduce contaminants into the surrounding waters [21]. In a well-flushed system, these contaminants might be dispersed, but many marinas that are surrounded by an artificial breakwall are specifically designed to restrict water flow [22,23]. This often results in a higher localised impacts as contaminants become bound to the sediments and localised effects to ecological communities [24,25].

Sediment ecology assessments provide a useful tool to monitor and detect negative effects of environmental changes associated with boating infrastructure impacts. Benthic infaunal communities have been used as bioindicators of sediment health due their sensitivity to changes in contaminant concentrations in sediments, through ingestion, and uptake via pore water [26]. A considerable amount of literature exists on impacts to the composition of sediment infauna communities in ‘hotspots’ of contamination in coastal and estuarine systems [5,27,28]. However, rarely have these studies considered how the design and spatial allocation of infrastructure developments might reduce the future spatial extent of these impacts within marine parks.

We evaluated the capacity for spatial management of environmental stressors associated with boating infrastructure (marinas, boat ramps and jetties) in the Batemans Marine Park on the south coast of New South Wales (NSW), Australia (northern boundary = 35°31.086’S and southern boundary = 36°22.290’S). This ~850 km^2^ marine protected area encompasses all tidal waters from the mean high tide mark to the limit of state waters (ca. 3 nm from land), including numerous estuaries and coastal lagoons. The Marine Park is zoned into 4 types of areas: sanctuary zones, habitat protection zones, general use zones and special purpose zones, which represent 19.1%, 43.3%, 37.2% and 0.4% of the entire park, respectively. Several special purpose zones were set up specifically to relax marine park regulations in areas with significant existing foreshore and maritime infrastructure. These zones were designed to facilitate sustainable development of boating infrastructure in a geographically-limited area, reducing development pressure on more pristine areas of the marine park. We evaluated the environmental and ecological changes associated with different boating infrastructure (marinas, boat ramps and jetties) inside a special purpose zone relative to reference sites situated within habitat protection zones. Our study represents the first quantitative assessment of the effectiveness of special purpose zones for management of foreshore and maritime infrastructure in a multi-use marine park.

## Materials and Methods

### Site description

**Fig 1:**
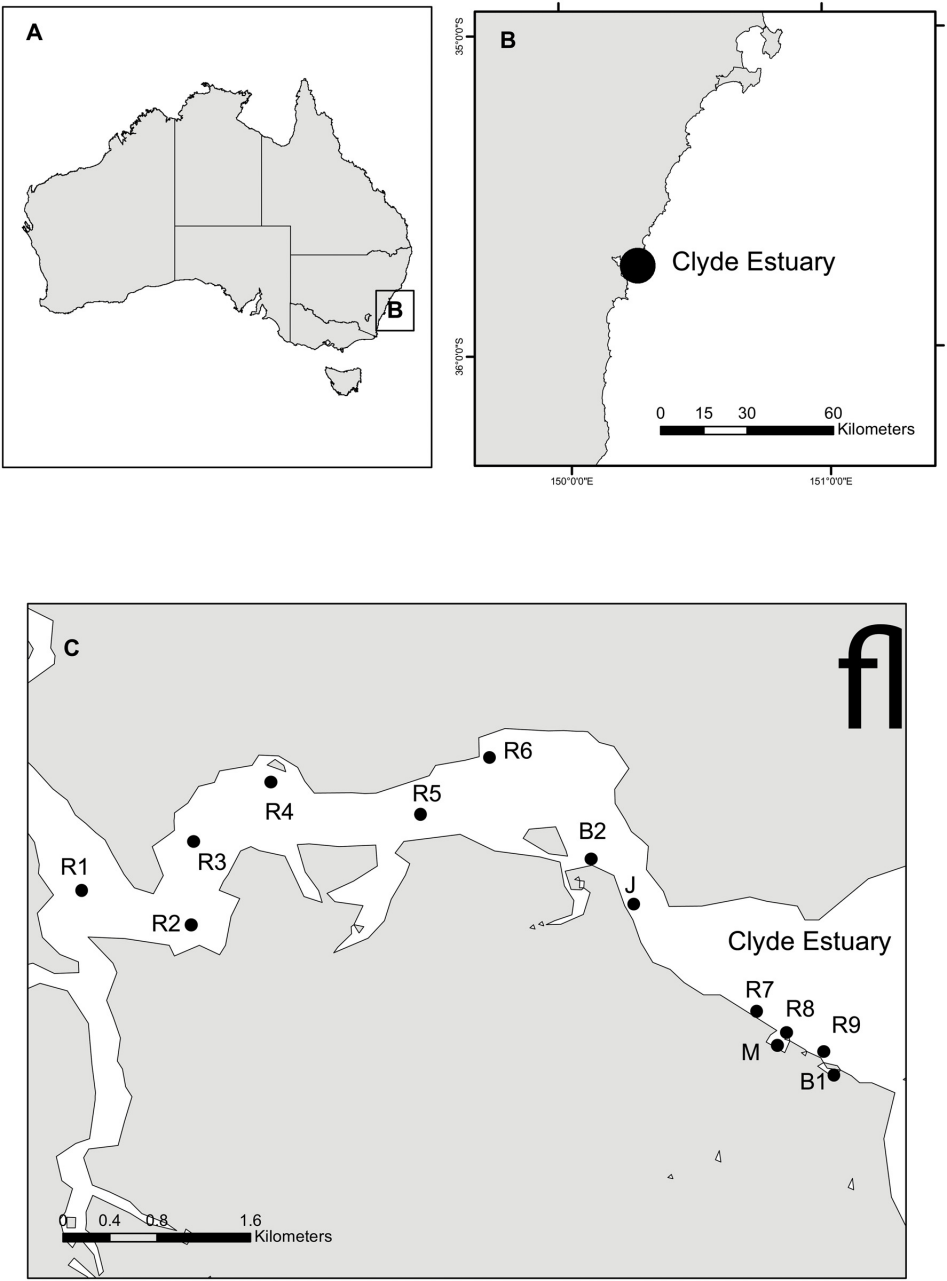
Map of the Clyde Estuary study sites located on the south east coast of Australia. Sites include boating structures (B1—boat ramp 1, M—marina, J—jetty, B2—boat ramp 2) reference sites (R1-R9). All boating structures and reference sites R7-R9 were located within the designated special purpose zone. Reference sites R1-R6 were located within the habitat protection zone of Batemans Marine Park.

Sediments were collected from multiple sites from the Clyde Estuary (entrance 35° 42.310'S, 150° 10.797'E) in the Batemans Marine Park (Fig 1). The Clyde Estuary has an area of ~17.5 km^2^ and a catchment area of ~1720 km^2^. The catchment is predominantly forested, with ~47% and ~44% of the catchment area protected in National Parks/Reserves and in State Forests, respectively [29]. The Clyde Estuary is largely undeveloped, and has marine park zoning of either habitat protection or sanctuary zone. There are several foreshore settlements in the lower catchment, with the largest being Batemans Bay. The estuarine area adjacent to this township has marine infrastructure including jetties, seawalls, a marina, several boat ramps and moorings. Given the existing foreshore development at Batemans Bay, 0.29 km^2^ of estuarine waters directly adjacent to the town was declared a special purpose zone to provide for foreshore and maritime facilities and infrastructure.

The marina studied was built approximately 25 years ago on previously undeveloped land and currently contains 126 wet berths for boats 4–8 m long. There are plans for expansion to more than double the size of the marina with the addition of 104 wet berths and multipurpose buildings such as offices, workshops and cafes and bars. Such projects and expansions are a common feature of estuarine shorelines, making it difficult minimise anthropogenic impacts on marine ecosystems.

### Survey design

To investigate the physico-chemical and biotic changes in sediment habitats associated with recreational boating structures in the Batemans Bay special purpose zone, we collected sediments adjacent to four boating structure and nine reference sites. Boating structure sites were adjacent to a marina (M), two boat ramps (B1 & B2) and a jetty (J) (Fig 1). The marina is surrounded by a breakwall with a central access point to the main channel. The boat ramp B1 has two lanes for vessel launch and retrieval and is also surrounded by a breakwall. Boat ramp B2 has a single lane with no breakwall. Boating structures were constructed of wood (J) or concrete (B2), or wood and concrete (M & B1). Reference sites (R1–R9) were located upstream and downstream of boating structure sites to account for the natural estuarine salinity gradient (Fig 1). Upstream reference sites were located near oyster leases, which limited boating activities in these areas. Downstream reference sites were in a well-flushed boating channel, but no anchoring or mooring was permitted.

### Sample collection

Benthic sediment samples were collected between February and July 2012. Plasticware used in sediment collection was previously soaked in 5% HNO_3_ for a minimum of 24 h and then rinsed in deionised water (Milli-Q, 18 MΩ.cm) [30]. Four sediment grabs were collected at each site from 5 m depth using a Van Veen grab (250 cm^3^) to target surficial sediments (~top 3 cm). Grab sediments were homogenised in a clean tray and sub-samples were taken for infauna (500 mL) and analyses of metals (total) and grain size [31]. Infauna were preserved immediately following sampling with a mixture of 7% formalin in seawater and stained prior to preservation with Rose Bengal. Samples for metals and grain size analyses were kept in the dark on ice for transport and then stored frozen at -18°C. Temperature and salinity were measured at each site using a YSI-Sonde 6600v2 (Yellow Springs, USA) during sediment collection at ~5m depth.

### Sample processing

Metal concentrations of Cu, Pb and Zn were determined using a low-pressure aqua regia microwave digest based on USEPA method 3051A [32]. Specifically, the sample was thawed and a homogenised sub-sample of 5–10 g weighed out and dried overnight in a drying oven at 60°C. When dry, samples were manually ground with a mortar and pestle, which was rinsed with deionised water in between samples to prevent cross contamination. Approximately 0.3 g of the sample was then weighed out for the microwave-assisted acid-digestion. Six mL of concentrated nitric acid (Tracepur, Merck) and 2 mL of concentrated hydrochloric acid (Tracepur, Merck) was added to each sample. Samples were microwave digested for a period of 90 min at 80°C. Once cool, deionised water was added to each digest vessel, which was then repeatedly inverted to ensure contents were homogenised and allowed to settle overnight. The settled sample was decanted with approximately 20–30 mL of the digest supernatant poured into an acid washed polycarbonate tube. The supernatant was diluted to a 1:10 ratio of sample to deionised water respectively. The supernatants of each sample were diluted an additional 5 times prior to further analysis.

Total sediment metal concentrations in acid digests were determined using Inductively Coupled Plasma–Atomic Emission Spectroscopy (ICP-AES) (Perkin Elmer, Optima 7300DV, USA). The instrument was calibrated with matrix matched standards and certified reference material (CRM) PACS-2 (National Research Council, Canada) was analysed with every microwave digest and/or with every 8 samples in the ICP-AES for quality control. Two blanks were included in each microwave digest.

Infaunal samples were stained with Rose Bengal and preserved in 7% formaldehyde (Univar, Canada). 250 mL samples were passed through 2 mm (to remove large debris) and 500 μm sieves (to collect organisms) and then preserved in 70% ethanol (Chem Supply Pty Ltd, Australia) until identification. Organisms were sorted under a dissecting microscope and identified in most cases to species for polychaetes and family or order for other taxa present. Identifications were confirmed with Dr Pat Hutchings, Australian Museum and a reference collection stored at the University of New South Wales.

Sediment composition samples were wet sieved and separated in to three size fractions, as follows: Gravel (2 mm), sand (2 mm–63 μm) and fines (<63 μm). Each fraction was weight weighed and oven dried for 24 h at 60°C. Dried samples were weighed to determine the percentage contribution of each fraction.

### Data collection and analysis

Analyses of the effects of boating infrastructure included two factors: Location (Lo) and Site (Si). Location was treated as a fixed factor with two levels described: boating structure and reference. Site was a random factor with 4 boating structure sites and 9 reference sites nested within location.

Principal co-ordinates analysis (PCO) plots were used to visualise differences in 1) sediment infaunal communities and 2) the metal contaminant concentrations (normalised to the percentage of sediment fines, % <63μm) and physico-chemical variables (fines, salinity and temperature) among locations. Contaminant concentrations, sediment composition and physico-chemical variables are included in S1 Table. Differences in the infaunal community composition between boating structure and reference sites were investigated with permutational multivariate analyses of variance (perMANOVA) using untransformed data because perMANOVA is robust to heterogeneous variances and non-normal distributions [33]. Infauna abundance data are included in S2 Table. Analyses were performed on Bray-Curtis similarity matrices (Bray and Curtis 1957). Taxa abundance, diversity and individual taxa that explained the most variation in the infaunal community composition among locations (r > 0.3, number of individuals sampled > 1) were selected for univariate permutational ANOVA [33]. Data were untransformed and the analyses were run using Euclidean distance matrices. Differences in metals and physico-chemical variables between boating structure and reference sites were also investigated with univariate permutational ANOVA. Total sediment metal concentrations (Cu, Pb, Zn) investigated using raw values and values that were normalised to particle size (by dividing the respective metal concentrations by the percentage fines, % <63μm). Similarity matrices were constructed using Euclidean distance. Total metal concentrations were compared with the Sediment Quality Guidelines Values (SQGVs) [34,35].

All results were considered significant if *P* < 0.05. All analyses were conducted using PRIMER (Plymouth Routines in Multivariate Ecological Research) statistical software (v 6.1.11 PRIMER-E Ltd, UK).

### Ethics Statement

This study was carried out with the approval of the manager of Batemans Marine Park. All sediment and infaunal samples were collected from the Clyde Estuary in Batemans Marine Park Australia (35° 42.310'S, 150° 10.797'E) under Fisheries New South Wales collecting permits (P09/0072-2.1 & OUT12/389). The invertebrate species collected are not currently listed as endangered or protected.

## Results and Discussion

### Environmental and ecological changes associated with boating infrastructure

**Fig 2:**
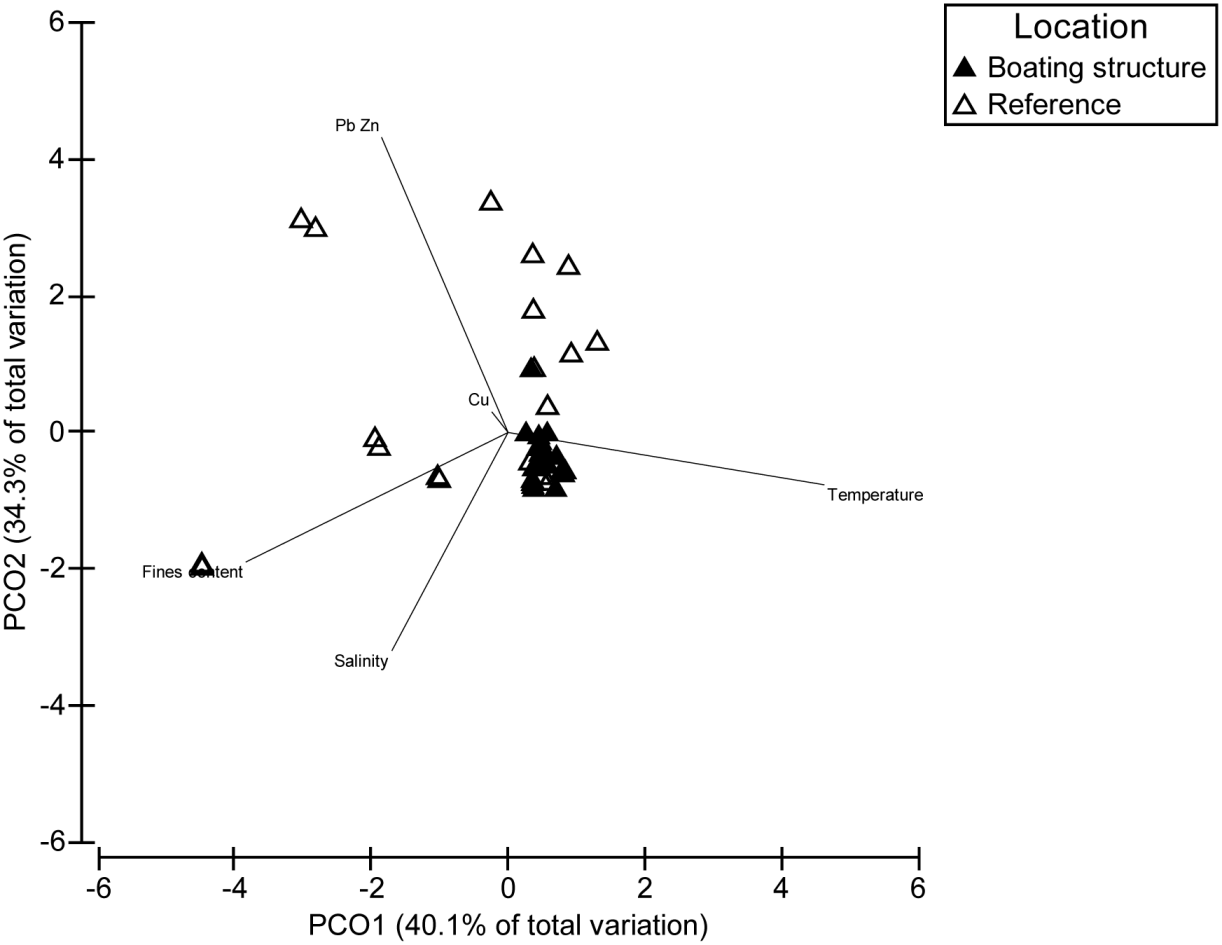
Multivariate visualisation of contaminant concentrations and environmental variables in Batemans Marine Park. PCO of contaminant concentrations (normalised to the percentage of sediment fines) and environmental variables using Euclidean similarity resemblance matrix.

Differences among reference sites were related to natural physico-chemical variables including fines content, salinity and temperature (Fig 2). Boating structure sites clustered more closely than reference sites suggesting more uniform physico-chemical conditions (Fig 2). The concentrations of lead and zinc were the main variables contributing to the differences between the boating structure and reference sites (Fig 2).

**Fig 3:**
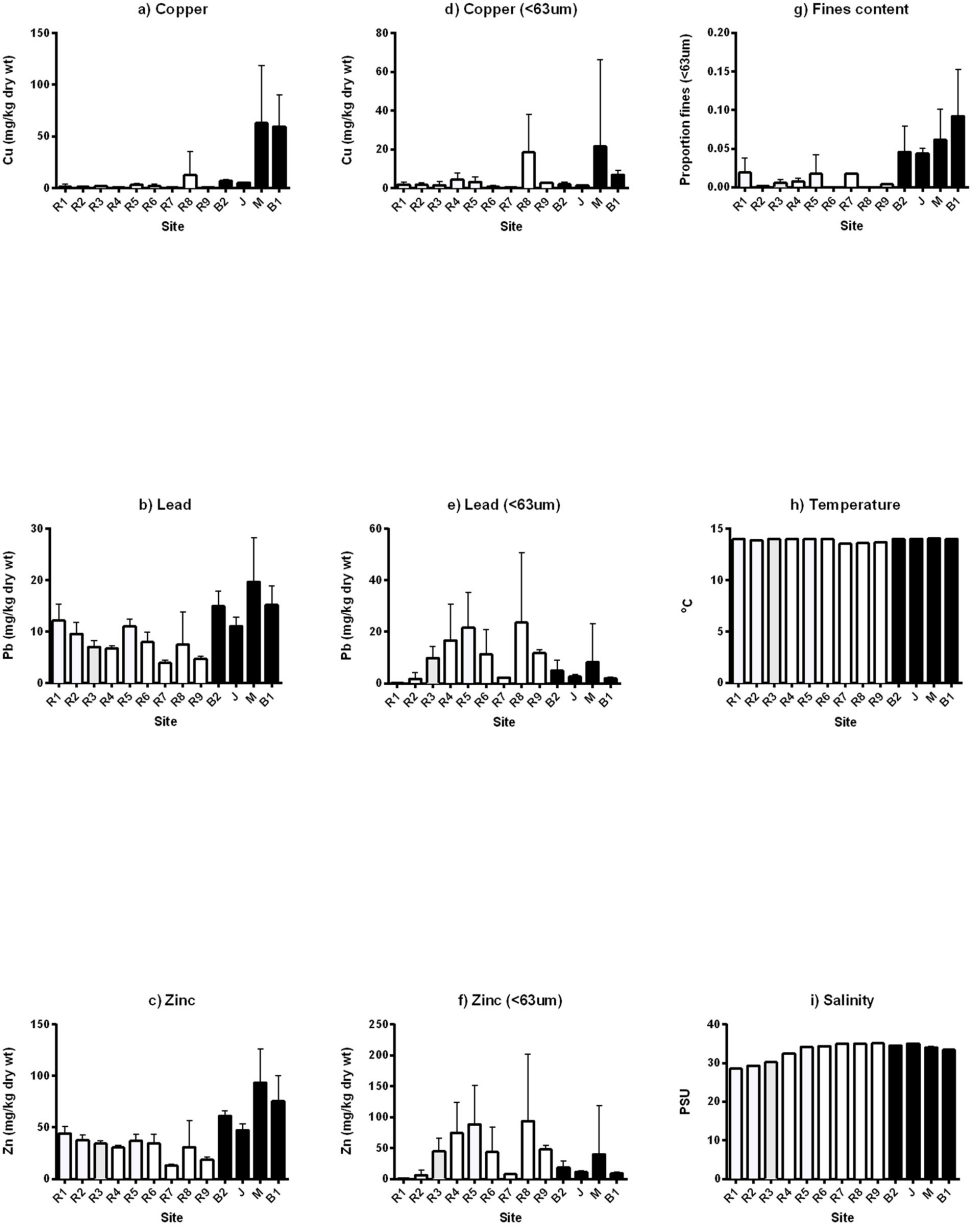
Contaminant concentrations and environmental variables in Batemans Marine Park. Mean (+/-S.E.) metal concentrations (μg/g) are shown for a) copper, b) lead, c)zinc and mean (+/-S.E.) d) silt content (% < 63 μm), e) temperature and f) salinity comparing boating structures within a special purpose zone to reference sites within special purpose and habitat protection zones in Batemans Marine Park. Sites include boating structures (B1—boat ramp 1, M—marina, J—jetty, B2—boat ramp 2) reference sites (R1-R9). White bars are reference sites and black bars are boating structures.

Boating infrastructure and activities are a major source of metal contaminants in coastal and estuarine systems [23,36,37]. Contamination of sediments with Cu, Pb and Zn is common in many environments, e.g. lead from historical use of leaded fuels, zinc from galvanised steel, and copper increasingly through passive leaching of antifouling paints [37,38] and the deposition of less bioavailable antifouling paint particles [39,40]. Furthermore, the treatment of wooden pilings in marinas and jetties with copper chromated arsenate (CCA) has been found to be a significant source of copper contamination to sediments [41]. In the current study, sediments adjacent to boating structures were more metal contaminated (Cu, Pb, Zn) than reference sites (Table 1a–1c, Fig 3a–3c). However, Cu, Pb and Zn concentrations were mostly below the Australian SQGVs of 65, 50 and 200 mg/kg, respectively [34,35] and therefore it is likely that these sediments present a low risk to the communities living in them [2,42]. Metal concentrations only exceeded SQGVs inside boating structures enclosed by a breakwall (B1 and M, Fig 3a–3c) suggesting potential negative ecological effects. However, changes to sediment communities were also observed at boating structures where SQGVs were not exceeded, but where fines content differed. When metal concentrations were normalised to the fines content in the sediments (Fig 3d–3f) the differences between boating structures and reference sites were no longer significant (Table 1d–1f). This suggests that some of the observed variation in metal concentrations can be explained by changes in the sediment physical composition. The accumulation of silt around boating structures rather than metals may be contributing more to the observed differences in the sediment communities.

**Table 1:** Results from permutational univariate ANOVA of metal contaminants and sediment composition sampled from sites adjacent to boating structures and reference sites.

Source	df	MS	Pseudo-F	P(perm)	MS	Pseudo-F	P(perm)	MS	Pseudo-F	P(perm)
		*a) Copper*			*d) Copper (<63um)*			*g) Fines content*		
Location	1	4.83	5.11	**0.012**	0.02	0.18	0.765	2.66	0.60	0.734
Site (Lo)	11	0.87	1.15	0.341	0.15	1.81	0.137	4.01	388.60	**<0.001**
Res	35	0.76			0.08			0.01		
		*b) Lead*			*e) Lead (<63um)*			*h) Temperature*		
Location	1	9.07	13.01	**0.005**	3.07	3.58	0.092	10.44	3.99	0.056
Site (Lo)	11	0.63	0.98	0.472	0.79	1.48	0.202	2.37	60.65	**<0.001**
Res	35	0.65			0.53			0.04		
		*c) Zinc*			*f) Zinc (<63um)*			*i) Salinity*		
Location	1	11.56	13.84	**0.002**	2.42	3.53	0.092	7.65	1.87	0.238
Site (Lo)	11	0.76	1.64	0.128	0.63	1.54	0.165	3.69	189.56	**<0.001**
Res	35	0.46						0.02		

Metal concentrations are presented as raw values (a-c) and normalized to the proportion of fines in the sediment (d-f) (dry wt mg/kg).

**Bold** p-values are significant α = 0.05.

We found patterns of increased fines content in sediments adjacent to boating structures (Fig 3g). These differences were only significant for some sites within locations (Table 1g), and interestingly did not extend beyond the marina breakwall to nearby reference sites (R7-R9, Fig 3g). As boating structures accommodate and protect vessels and are therefore designed to reduce flow [21], the higher deposition of fine sediments around boating structures compared to reference sites was not unexpected. Detrital deposition from epifaunal organisms growing on the structures is likely to have contributed to the observed organic enrichment [43]. Changes to flow and increased fines deposition have also been linked to increased contamination due to the greater affinity and capacity of fine sediments that bind contaminants [25,35,44]. Temperature and salinity also varied between sites within locations following an estuarine gradient, but did not differ between boating structures and reference sites (Table 1h and 1i, Fig 3h and 3i) so were unlikely to be driving the observed patterns.

**Fig 4:**
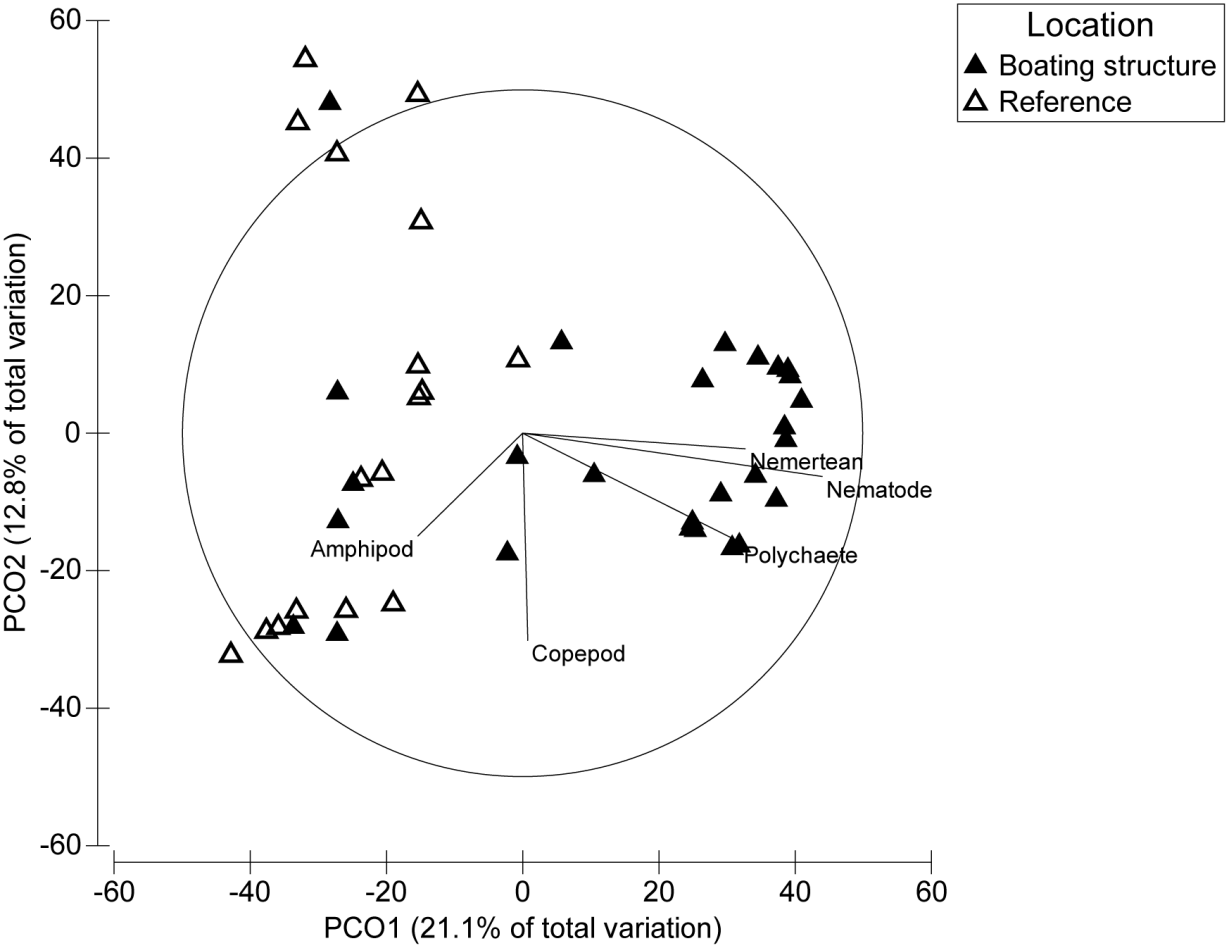
Multivariate visualization of sediment infauna assemblages in Batemans Marine Park. PCO of sediment infauna assemblages using Bray-Curtis similarity resemblance matrix constructed from fourth-root transformed biological data.

**Fig 5:**
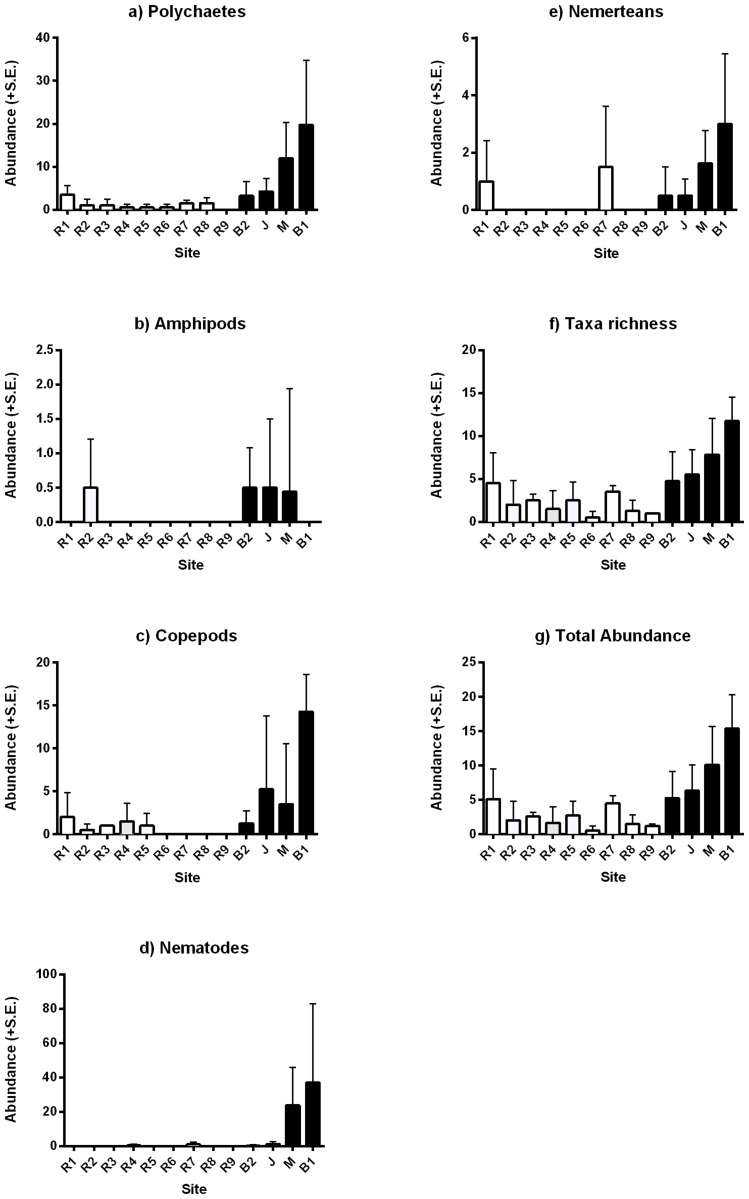
Sediment infauna abundance and diversity in Batemans Marine Park. Mean abundances (+/-S.E.) of (a) Polychaetes, (b) Amphipods, (c) Copepods, (d) Nematodes, (e) Nemerteans and (f) Taxa richness and (g) Total abundance comparing boating structures within a special purpose zone to reference sites within special purpose and habitat protection zones in Batemans Marine Park. Sites include boating structures (B1—boat ramp 1, M—marina, J—jetty, B2 —boat ramp 2) reference sites (R1-R9). White bars are reference sites and black bars are boating structures.

Sediment composition is a major factor that influences the colonisation of sediment organisms and structures infaunal communities [5,45,46]. We found that community composition in finer sediments adjacent to boating structures consistently differed from communities at the coarser reference sediments (Table 2a and Fig 4). Abundances of several individual taxa including polychaetes, copepods, nematodes and nemerteans were elevated in sediments near boating structures (Table 2b,2d–2f, Fig 5a,5c–5e). Amphipods showed similar patterns, but abundances were generally low and variable among sites within locations (Table 2c, Fig 5b). Furthermore, the magnitude of difference appeared to be affected by the design of the structure. Enclosing boating structures with a breakwall was associated with the greatest differences from reference sites (e.g. marina and boat ramp B1) (Fig 5). Despite moderately elevated concentrations of copper, we observed greater taxa richness and overall abundance inside the boat ramp and marina areas (Table 2g–2i), Fig 5f and 5g). Although the correlative nature and scale of this study cannot separate the effects of metals and fines content on sediment infauna, increased richness and abundance of organisms is unlikely to be a toxic effect of metals, but may be related to both the increased deposition of organic material suggested by higher fines content [47] and an influx of tolerant opportunistic species such as capitellid polychaetes. Previous lab and field studies have highlighted that capitellids often dominate disturbed environments and are tolerant of chemical disturbances from increased organic enrichment [5,47], environmental stress [48,49] and metal contamination [50].

**Table 2:** Results from permutational multivariate ANOVA of infauna community and univariate ANOVA of individual taxa and taxa richness and abundance sampled from sites adjacent to boating structures and reference sites.

Source	df	MS	Pseudo-F	P(perm)	MS	Pseudo-F	P(perm)
		*a) Community composition*			*b) Polychaetes*		
Location	1	13169	2.26	**0.049**	730.60	9.65	**<0.001**
Site (Lo)	11	5378	1.89	**<0.001**	68.87	1.33	0.262
Res	35	2852			51.68		
		*c) Amphipods*			*d) Copepods*		
Location	1	0.26	1.44	0.205	281.98	6.37	**0.002**
Site (Lo)	11	0.24	0.21	0.925	40.41	1.36	0.226
Res	35	1.18			29.63		
		*e) Nematodes*			*f) Nemerteans*		
Location	1	2300	5.37	**<0.001**	12.33	5.51	**0.015**
Site (Lo)	11	394	1.01	0.449	2.05	1.48	0.199
Res	35	390			1.38		
		*g) Taxa richness*			*h) Total abundance*		
Location	1	273.52	18.57	**<0.001**	8988	7.32	**0.002**
Site (Lo)	11	13.36	1.21	0.323	1120	1.27	0.292
Res	35	11.05			885		

Other taxa were omitted from ANOVA analyses due to absence from a location.

**Bold** p-values are significant at α = 0.05.

### Spatial planning considerations for special purpose zones in multi-use marine parks

A key objective of the special purpose zone in the Clyde Estuary was to provide for environmentally-responsible foreshore and maritime facilities and infrastructure geographically limiting potential impacts to more pristine parts of the Batemans Marine Park. Our results suggest that there are important ecological changes in sediment communities adjacent to boating structures, and that these changes occur where organic deposition and contamination is increased around structures. However, these changes were only observed within the special purpose zone in close proximity to the boating structures. Therefore the spatial allocation to infrastructure appears to currently be effective at enabling multiple activities within Batemans Marine Park without negative ecological consequences to the surrounding sanctuary and habitat zones. The sampling design was constrained by the limited number of boating structures in the Clyde Estuary and the fact they varied in age, construction material and maintenance schedule. This variation could have contributed to the high variability in adjacent sediment conditions. Given these limitations, future studies could improve the generality of findings by sampling boating structure in more multi-use marine parks estuaries in southern NSW.

Furthermore, the design of infrastructure inside these zones appears to go some way to constraining the extent of any effects from anthropogenic activities. For example environmental changes associated with the marina in the Clyde River were constrained to within the extent of the breakwalls. Our study was limited to two boating structures that were enclosed by breakwalls and future work should investigate a larger sample size of marinas and boating structures with/without breakwalls to investigate the potential for breakwalls more generally to trap contaminants and prevent spread. However, this could create a delicate trade-off since if impacts are concentrated inside breakwalls and result in hotspots of contamination and invasive species occurrence in proximity to transport vectors [22]. Boating structures such as marinas that can become extensive in size and the number of vessels supported would likely create greater risk within marine parks. Careful planning of the size and location of boating infrastructure is therefore crucial in any spatial planning strategy for marine parks.

## Conclusion

Boating infrastructure changes local environmental conditions and we found increased fines and moderate metal concentrations in proximity to the marina, jetty and two boat ramps within the special purpose zone of the Clyde Estuary in Batemans Marine Park. Sediment faunal assemblages also changed significantly adjacent to these boating structures compared to reference sites. However, these environmental and ecological changes were only observed within the special purpose zone of the marine park and effects did not extend to reference sites. This study highlights how special purpose zoning in a multi-use marine park can enable sensible, sustainable development in areas with existing intensive use and infrastructure, while ensuring other more natural areas can be managed more vigorously for conservation of biodiversity.

## Supporting Information

S1 File Financial Support Statement. (DOCX)

S1 Table Metal (ug/g dry wt) and PAH contaminant concentrations (mg/kg dry wt), total organic carbon (TOC) and sediment composition (% fines < 63um) measured in benthic sediments. Sites include boating structures (B1—boat ramp 1, M—marina, J—jetty, B2—boat ramp 2) reference sites (R1-R9). All boating structures and reference sites R7-R9 were located within the designated special purpose zone. Reference sites R1-R6 were located within the habitat protection zone of Batemans Marine Park.
(XLSX)

S2 Table Infauna abundances sampled from benthic sediments. Sites include boating structures (B1—boat ramp 1, M—marina, J—jetty, B2—boat ramp 2) reference sites (R1-R9). All boating structures and reference sites R7-R9 were located within the designated special purpose zone. Reference sites R1-R6 were located within the habitat protection zone of Batemans Marine Park.
(XLSX)
