# Supplementary material for: Sediment Contaminants and Infauna Associated with Recreational Boating Structures in a Multi-Use Marine Park
Source: PLoS One. 2015 Jun 18;10(6):e0130537. doi: 10.1371/journal.pone.0130537 (PMC4472803; doi:10.1371/journal.pone.0130537)
Supplement: S1 File — (DOCX) [file pone.0130537.s001.docx]

This research was primarily supported by the Australian Research Council through an Australian Research Fellowship awarded to Johnston and a Linkage Grant LP0990640 awarded to Johnston and Kelaher.
